# Relationships between Functional Movement Quality and Sprint and Jump Performance in Female Youth Soccer Athletes of Team China

**DOI:** 10.3390/children9091312

**Published:** 2022-08-29

**Authors:** Junjie Zhang, Junlei Lin, Hongwen Wei, Haiyuan Liu

**Affiliations:** 1 Graduate School, Capital University of Physical Education and Sports, Beijing 100091, China; 2 School of Strength and Conditioning Training, Beijing Sport University, Beijing 100084, China

**Keywords:** youth, sprint, jump, YBT, FMS

## Abstract

This study aimed to determine the optimal functional movement screen (FMS) cut score for assessing the risk of sport injury, and to investigate the correlations between functional movement quality and sprint and jump performance. Twenty-four (N = 24) athletes performed all tests in one day at 10–30 min intervals, and the FMS test was performed first, without a warm-up session. After a standard warm-up, athletes then completed the Y-balance Test (YBT), sprint, counter-movement jump (CMJ), and standing long jump (SLJ), in turn. For each test, the best of three attempts was recorded for further analysis. A receiver operating characteristic (ROC) curve and area-under-the-curve (AUC) were used to determine the optimal FMS cut score for assessing the risk of sport injuries, and Spearman’s rank correlation analysis was used to quantify associations between functional movement scores and athletic performance. The average FMS score was 16.2 and the optimal FMS cut score for assessing the risk of sport injuries was 14.5. There were moderate relationships between total FMS score and 10–20 m sprint time (r = −0.46, *p* < 0.05), between In-line Lunge and 0–20 m sprint time (r = −0.47, *p* < 0.05), between Shoulder Mobility and 0–10 m sprint time (r = −0.48, *p* < 0.05), and between Trunk-stability Push-up and 10–20 m sprint time (r = −0.47, *p* < 0.05). Moreover, Hurdle Step score was largely correlated with 0–10 m time (r = −0.51, *p* < 0.05). For Y-balance, moderate correlations were observed between CMJ height and anterior asymmetry score (r = −0.47, *p* < 0.05) and posteromedial asymmetry score (r = −0.44, *p* < 0.05). However, there were no significant associations between YBT performance (asymmetric in three directions and composite score) and sprint performance (*p* > 0.05). Taken together, the results indicate that a FMS score of 14 is not a gold standard for assessing the risk of injury in all populations; we recommend that the FMS cut score of 14.5 should be the optimal score for assessing risk of injury in young female elite soccer players. Moreover, the FMS and YBT were introduced to assess the quality of functional movements, and they cannot be used to assess sprint and jump performance. Practitioners can use components of the FMS that have similar characteristics to specific sports to assess athletic performance.

## 1. Introduction

The game of professional soccer demands a high level of physical performance from players, all of whom need to have excellent strength, speed, endurance, etc. For soccer athletes, several tests have been conducted to assess performance. The most common measurement tools include 1-repetition maximum (1RM) [1], rate of force development [2], 505 change of direction [3], and the yo-yo test [4]. Notably, these tests only provide information on musculoskeletal and cardiopulmonary fitness, and lack functional insight. Insufficient functional ability can reduce athletic performance and increase the risk of injury; it is thus essential to identify functional deficits using functional screening tools [5].

Presently available functional screening tools include functional movement screening (e.g., functional movement screen, FMS) and functional asymmetric screening (e.g., Y-balance Test, YBT). FMS was introduced to evaluate functional limitations, and was essential in determining mobility and stability when performing fundamental movement skills [6]. It can also assess maximal strength, postural control, and range of motion [7]. The capability of functional movement refers to the ability to perform essential motor skills (locomotor, stabilization, and manipulation) under controlled conditions [6]. The FMS test comprises seven distinct tests, which are scored from 0 to 3, and a composite score ranges from 0 to 21. Furthermore, FMS is a reliable assessment tool for interrater (ICC: 0.81; 95% CI: 0.74–0.8) and interrater reliability (ICC: 0.81, 95% CI: 0.70–0.92) [8]. YBT was derived from the Star Excursion Balance Test (SEBT) [9] and can be used to assess dynamic balance, functional asymmetric, proprioception, and strength [10]. Numerous studies have proved that YBT is a reliable assessment of dynamic balance (ICCS: 0.85–1.00) [9,11].

Due to their role in identifying functional deficits, these functional screening tools can be a useful predictor of injury risk. Massive studies have examined the validity of the FMS as a tool for predicting injury risk. A study conducted by Kiesel et al. [12] found that football athletes with FMS score of ≤14 showed greater risk of sports injury than those with higher scores. Elsewhere, this finding was also proved by other studies [13,14]. However, FMS cut score is influenced by a variety of factors, including training state, sport participating, gender, age, etc. It is therefore necessary to determine the optimal FMS cut score for different populations, a number which can then provide useful information for athletes and practitioners. However, to the author’s knowledge, no studies have been conducted on the optimal FMS cut score in populations of young female football athletes.

These functional screening tools were introduced to assess movement quality, which also can be indicative of sports performance [15]. Okada et al. [16] found that the FMS score is relatively strongly associated with lower-limb strength performance (r = −0.38~−0.46) in recreational mixed-gender athletes. Similarly, significant correlations between FMS and vertical jump and agility have been observed in youth soccer athletes [17]. Furthermore, Liang et al. [18] explored whether FMS score was associated with athletic performance in collegiate baseball players. The authors reported that greater FMS scores indicated better speed and agility performance. However, conflicting results have been reported in other studies. Parchmann and McBride [19] reported no significant correlations between FMS scores and sprint time, jumping, or agility performance in college golfers. Similarly, the FMS score was not associated with selected physical performance in active subjects [20]. It is worth noting that the authors of these studies creatively combined athletes of different genders or studied male players only.

Therefore, this study aims to: (1) determine the optimal FMS cut score for assessing risk of sports injury in female youth football players; and (2) investigate the correlations between functional movement quality (FMS and YBT) and sprint and jump performance. It is hypothesized that the optimal FMS cut score is higher than 14, and that functional movement quality is strongly correlated with sprint time and jump performance.

## 2. Materials and Methods

A cross-sectional design was used to determine the optimal FMS cut score and whether the functional movement quality (FMS and YBT) was related to sprint and jump performance in young female soccer athletes. Subjects performed all tests in one day at 10–30 min intervals. The FMS test was conducted without warm-up. After the FMS test, all athletes underwent 20 min standard warm-up sessions, including 5 min cycling and 15 min static and dynamic stretch [21]. Afterward, the athletes completed Y-balance, sprint, and jump tests in turn. For all tests, all athletes took three attempts at 2–3 min intervals, and the best performance was recorded for further analysis [22].

### 2.1. Participants

Twenty-four female youth soccer athletes (age: 14.79 ± 0.40 years, height: 166.63 ± 5.29 cm, mass: 53.33 ± 6.19, BMI: 19.17 ± 1.71 kg/m^2^) from Team China participated in this study. Before this study, all athletes were provided information about the procedures and study objectives. Participants were excluded if they were in their menstrual cycle. This study was approved by the Ethics Committee of Beijing Sport University (14 January 2022), and all subjects voluntarily participated in this study and signed informed consent forms.

### 2.2. 20 m Sprint

The 20 m sprint was measured by the light timing system (Swift EZE Jump, Version 2.5.28, Brisbane, Australia). Three pairs of light gates were placed at 0, 10 and 20 m with a height of 60 cm. To avoid triggering the beam of the timing gates before the start of the test, subjects started 30 cm behind the starting line in a 2-point stance.

### 2.3. Vertical and Horizontal Jump

The countermovement jump (CMJ) was measured using a force plate form (Kistler, Version 2822A1-1, Winterthur, Switzerland). Subjects were instructed to rapidly squat down to a predetermined degree (approximately 60° knee flexion angle) and then jump as high as possible. The distance of the standing long jump (SLJ) was measured using a standard tape.

### 2.4. Functional Movement Screen

All athletes completed a functional movement assessment through the FMS test, which was performed according to the standard guidelines [23]. Athletes were not allowed to have any warm-up prior to the test. Each component of the FMS was scored from 0 to 3. The score of 0 indicated that participants felt pain during the test, and the score of 3 indicated perfect performance. Each of the seven movements was repeated no more than three times, and only the best of the three repeats was recorded.

### 2.5. Y-Balance Test

In YBT, the dynamic balance was examined in three directions: anterior, posteromedial, and posterolateral. The athlete stood on one leg in the stationary center of the Y-balance kit with hands on the hips, and was instructed to stretch the contralateral lower limb as far forward as possible. After three formal trials on one limb, the athlete stood on the opposite lower limb and stretched in the same direction. Each direction was repeated two times with 1 min interval, and the best repetition was recorded. In addition, the lower-limb length was measured from the athlete’s anterior superior spine to Epicondylar medial ankle [24,25]. YBT composite score was calculated using the following formula: (anterior + posteromedial + posterolateral performances in cm)/3 × lower-limb length in cm) × 100 [24].

### 2.6. Statistical Analysis

Data are presented as mean ± standard deviation (SD). The FMS cut score was determined using the receiver operating characteristic (ROC) curve and area-under-the-curve (AUC). Spearman’s rank correlation analysis was used to quantify associations between selected variables. The strength of correlation was determined as small (<0.29), moderate (0.3~0.49), large (0.5–0.69), or very large (>0.7) [26]. The statistical analysis was performed using SPSS (Version 22 for Windows, Armonk, NY, USA. IBM Corp). Statistical significance was set at *p* < 0.05.

## 3. Results

### 3.1. Descriptive Characteristics 

Table 1 demonstrates the descriptive statistics for characteristics of participants, functional screen, and physical performance. All athletes completed all five tests. The players’ positions were striker (*n* = 7), midfielder (*n* = 6), fullback (*n* = 8) and goalkeeper (*n* = 3). The FMS score ranged from 13.90 to 18.50, with a mean of 16.20 ± 2.30. Moreover, the mean YBT composite score was 96.65 ± 4.14, ranging from 92.51 to 100.79.

### 3.2. Receiver Operating Characteristic Curve and Area under the Curve Analyses 

For all athletes, the FMS cut score was 14.5. A relatively high AUC of 0.90 was observed (*p* = 0.004), and the sensitivity and specificity were 0.889 and 0.833, respectively. Moreover, 17 of the 24 athletes (70.8%) scored higher than 14.5.

### 3.3. Associations between FMS, Y-Balance and Sprint and Jumping Performance 

Moderate correlations were observed between FMS score and 10–20 m sprint time (r = −0.46, *p* < 0.05), between In-line Lunge score and 0–20 m sprint time (r = −0.47, *p* < 0.05), between Shoulder Mobility score and 0–10 m sprint time (r = −0.48, *p* < 0.05), and between Trunk-stability Push-up score and 10–20 m sprint time (r = −0.47, *p* < 0.05). Hurdle Step score was largely correlated with 0–10 m time (r = −0.51, *p* < 0.05). Moreover, SLJ and CMJ performance did not correlate with FMS score and its seven movements (*p* > 0.05). Moderate correlations were observed between CMJ height and Anterior Asymmetry score (r = −0.47, *p* < 0.05) and Posteromedial Asymmetry score (r = −0.44, *p* < 0.05). Nevertheless, there were no significant correlations (*p* > 0.05) between YBT performance (asymmetric in three directions and composite score) and sprint performance (0–10 m, 10–20 m and 0–20 m sprint time) (Table 2).

## 4. Discussion

To the best of our knowledge, this is the first study to determine the optimal FMS cut score for female youth football players, and the first to investigate the correlations between the quality of functional movement (FMS and YBT) and sprint and jump performance. This study found that the optimal FMS cut score was 14.5, with a sensitivity of 0.889 and Youden index of 0.722, which indicates a higher sensitivity and a lower false-positive rate of FMS in elite young female football players. Moreover, FMS score was moderately correlated with 10–20 m sprint time, but not with SLJ and CMJ performance. Moreover, significant relationships were also observed between In-line Lunge score and 0–20 m sprint time, between Shoulder Mobility score and 0–10 m sprint time, and between Trunk-stability Push-up score and 10–20 m sprint time, and between Hurdle Step score and 0–10 m time. There were no significant associations between YBT composite score and jump and sprint performance.

Massive studies have proved that FMS score has been significantly correlated with the risk of sport injuries in elite athletes [27]. A study conducted by Kiesel et al. [12] found that for elite American football athletes, the risk of non-contact injury was increased when the FMS score was below 14. However, elite youth soccer players with an FMS total score of >14 had less groin and hip dysfunction [28]. Notably, for different populations, an FMS score of 14 is not necessarily a gold standard. In the present study, using a score of 14.5 as the FMS cut score, the sensitivity is high, and the misclassification rate is low (16.7%). If the FMS cut score is 15.0, although the misclassification rate is 0, the Youden index is 0.611, which is less than 0.722, while the sensitivity of 0.611 is less than 0.889. Players with an FMS score of 14.5 have a more significant risk of injury than elite youth female football with an FMS > 15.0. Similarly, Zhao and Zhou [29] examined the FMS cut score in Chinese national team swimmers, and the authors reported an optimal FMS cut score of 16.5. Moreover, for elite fencers and shooters, the optimal FMS cut score was 15 [30]. These differences in optimal FMS cut score can be explained by several factors, including different training characteristics, competition requirements, sports of athletes participating, the presence of contact injuries, the sensitivity of the observation, the specificity, the chi-square values, the pre- and post-test values, etc. Therefore, the characteristics of the specific sport must be considered when using FMS score to assess the risk of sport injuries.

Much attention has been focused on whether the quality of functional movements can be a valid and effective method of assessing the athletic performance in elite players. Bennett et al. [31] examined the relationship between movement quality and physical performance and explored whether improved movement ability positively transferred to sport performance. The authors reported that FMS score was significantly and slightly associated with 5 m sprint time and agility performance. Moreover, significant increases in FMS score and speed performance were observed after one year, but there were no significant relationships between these improved variables. Similarly, no significant correlation was observed between FMS score and athletic performance in another previous study [32]. These findings were in line with the results of the present study, which found only a significant association observed between FMS score and 10–20 m sprint time. These results can be explained by several reasons. First, for each specific sport, the movement quality plays a small role compared to other factors that contribute to improved athletic performance. For example, speed and power output are the most critical factors for success in the 100 m sprint race [33,34]. Second, the FMS only reflects the level of selected isolated fundamental movement patterns, which compromises its predictive effects on capabilities such as execution speed, agility, etc. Third, the specific technical movement skill of force application is a determinant factor of sprint performance, rather than is fundamental movement [35]. The quality of specific technical skills affects the efficiency of force generation during sprinting. However, there are significant differences between fundamental movements skills and specific technical skills. Though the ability to perform the fundamental movement can influence the development of specific techniques skills to some extent, for elite athletes, specific technical skills are also influenced by cognitive, habitual, and other aspects. Therefore, FMS score cannot be used to predict performance in youth soccer players.

Specifically, in the present study, significant relationships were observed between sprint performance and In-line Lunge score, Trunk-stability Push-up score, Shoulder Mobility score, and Hurdle Step score. There are several similar factors in the development of these tasks (sprint and movements of FMS mentioned above) that could explain these findings. Both In-line Lunge and Hurdle Step tasks reflect the capability of total-body coordination and integration in horizontal orientation [16], these abilities are also required in sprint performance. Moreover, the Hurdle Step task assesses the abilities of knee and hip flexion, as well as single-leg postural control. These abilities also play a crucial role in sprint performance. Trunk-stability can also be referred to as core stability. Core stability helps to efficiently transfer the force through the kinetic chain and avoids “energy leak” during the sprint [36]. Shoulder Mobility impacts the arm movement during the sprint. The role of arm-leg coupling is to reduce the rotation of the body, and arm action may be essential to optimize the body position for ground force application during the sprint. When arm movement is restricted by other factors, for example, limited shoulder mobility, sprint performance will be compromised [37]. Therefore, this study suggests that it is considered reasonable to use components of FMS that have characteristics similar to a specific sport to assess specific performance, rather than looking to the FMS composite score.

In the present study, we found that there were no significant correlations between Y-balance composite score and sprint and jump performance. This finding was consistent with the result of a previous study [38], which found no significant relationships between YBT performance and vertical and horizontal jump. Similarly, the Y-balance test was introduced to assess the stability of the lower or upper limbs, and it plays a relatively small role in athletic performance. It is thus not appropriate for predicting athletic performance. In contrast, Bennett et al. [39] found significant correlations between YBT performance and physical performance variables, but the strength of correlation was small (r: 0.21~0.36). Moreover, many studies have reported that YBT performance was related to knee extensor strength [40], hip abduction strength [41], hip extension [41], and external rotation strength [41]. However, there is no direct evidence to suggest that improving hip and knee strength by increasing YBT score can transfer to athletic performance. Therefore, YBT composite score is also not suitable for assessing physical performance.

The strengths of this study included: (1) the subjects of this study were lite athletes who were familiar with valid running and jumping movement skills, which can avoid compromising the results of this study due to invalid movement skills; (2) excessive fatigue induced by high-intensity competition that would negatively affect the results of this study. But this study was conducted during pre-season, athletes without excessive fatigue can perform better, which can improve the accuracy of the results of this study. However, there are some limitations in this study. First, this study did not record the participants’ sports injuries, so we could not determine whether the FMS score can be used to predict the risk of sport injuries. Second, this study only included 24 players, the sample size is small, which may compromise the accuracy of the results. Therefore, future studies should include more participants. Third, this study only included female youth soccer players, and did not study male youth athletes. Therefore, the results of this study may be limited to male youth athletes.

## 5. Conclusions

In conclusion, a FMS score of 14 is not the gold standard for assessing risks of injury in all populations; this study has suggested that the optimal FMS cut score for assessing the risk of sport injury is 14.5 in female youth elite football players. Moreover, the FMS and YBT were introduced to screen for assessing injury risk, and neither test’s composite score is appropriate for assessing physical performance. We suggest that practitioners can instead use components of the FMS that have characteristics similar to specific sports to assess athletic performance.

## Figures and Tables

**Table 1:** Descriptive statistics for characteristics of participants, functional screening, and physical performance.

Variables (Mean ± SD)	Total (*n* = 24)	Striker (*n* = 7)	Midfielder (*n* = 6)	Fullback (*n* = 8)	Goalkeeper (*n* = 3)
Age (years)	14.79 ± 0.40	14.42 ± 0.49	15.00 ± 0.00	15.00 ± 0.00	14.67 ± 0.47
Height (cm)	166.63 ± 5.29	162.57 ± 3.89	165.50 ± 3.54	167.50 ± 3.35	176.00 ± 1.63
Mass (kg)	53.33 ± 6.19	50.00 ± 5.63	55.33 ± 8.13	54.00 ± 4.30	55.33 ± 3.39
BMI (kg/m^2^)	19.17 ± 1.71	16.86 ± 1.43	20.11 ± 2.11	19.22 ± 1.13	17.87 ± 1.21
FMS score	16.20 ± 2.30	16.14 ± 1.25	16.50 ± 2.36	15.87 ± 2.80	16.70 ± 2.49
YBT score	96.91 ± 4.00	98.15 ± 3.58	96.66 ± 4.18	95.99 ± 4.23	95.55 ± 4.7
0–10 m (s)	2.06 ± 0.05	2.08 ± 0.05	2.07 ± 0.04	2.05 ± 0.06	2.04 ± 0.02
10–20 m (s)	1.45 ± 0.05	1.47 ± 0.07	1.44 ± 0.03	1.44 ± 0.03	1.47 ± 0.04
0–20 m (s)	3.52 ± 0.89	3.55 ± 0.12	3.53 ± 0.7	3.49 ± 0.07	3.51 ± 0.06
CMJ (cm)	29.62 ± 3.62	30.07 ± 2.80	28.32 ± 4.38	30.79 ± 3.88	28.00 ± 1.24
SLJ (cm)	208.17 ± 10.60	208.14 ± 7.47	199.67 ± 8.05	210.63 ± 10.96	218.67 ± 6.94

BMI, Body Mass Index; FMS, Functional Movements Screen; YBT, Y-Balance Test; CMJ, Countermovement Jump; SLJ, Standing Long Jump; *n*, number; years, years; cm, centimeter; m, meter; s, second; kg, kilogram.

**Table 2:** Associations between FMS and athletic performance (r, 95% CI).

Tests	0–10 m	10–20 m	0–20 m	SLJ	CMJ
FMS Score	−0.29	−0.46 *	−0.35	0.20	−0.13
	(−0.65, 0.13)	(−0.76, −0.04)	(−0.66, 0.06)	(−0.29, 0.64)	(−0.56, 0.36)
Deep Squat	0.10	−0.24	0.06	0.17	−0.29
	(−0.36, 0.57)	(−0.68, 0.22)	(−0.39, 0.49)	(−0.29, 0.58)	(−0.70, 0.25)
Hurdle Step	−0.51 *	−0.01	−0.40	0.32	0.19
	(−0.75, −0.17)	(−0.44, −0.38)	(−0.68, −0.02)	(−0.18, 0.70)	(−0.26, 0.63)
In-line Lunge	−0.38	−0.37	−0.47 *	0.27	0.03
	(−0.75, 0.07)	(−0.71, 0.03)	(−0.80, −0.06)	(−0.23, 0.71)	(−0.45, 0.51)
Shoulder Mobility	−0.48 *	−0.13	−0.37	0.10	0.14
	(−0.80, −0.08)	(−0.53, 0.27)	(−0.70, 0.02)	(−0.34, 0.56)	(−0.29, 0.54)
ASLR	0.14	0.21	0.14	−0.14	−0.15
	(0.01, 0.35)	(0.11, 0.44)	(0.00, 0.33)	(−0.35, 0.00)	(−0.39, −0.02)
TSPU	−0.27	−0.47 *	−0.33	0.35	−0.07
	(−0.66, 0.17)	(−0.79, −0.08)	(−0.71, 0.12)	(−0.06, 0.67)	(−0.50, 0.35)
Rotary Stability	−0.02	−0.12	−0.06	−0.20	0.23
	(−0.21, 0.17)	(−0.33, 0.02)	(−0.26, 0.09)	(−0.45, −0.08)	(0.11, 0.46)
Anterior (%)	0.35	−0.10	0.10	−0.29	−0.47 *
	(−0.11, 0.69)	(−0.49, 0.30)	(−0.36, 0.49)	(−0.67, 0.15)	(−0.72, −0.10)
Posteromedial (%)	0.13	−0.05	0.15	−0.02	−0.44 *
	(−0.32, 0.56)	(−0.44, 0.30)	(−0.26, 0.51)	(−0.47, 0.41)	(−0.72, −0.06)
Posterolateral (%)	0.40	0.02	0.28	−0.16	−0.13
	(−0.06, 0.69)	(−0.41, 0.44)	(−0.17, 0.62)	(−0.60, 0.38)	(−0.57, 0.33)
YBT Score	−0.22	−0.19	−0.29	0.11	−0.30
	(−0.58, 0.27)	(−0.60, 0.28)	(−0.64, 0.18)	(−0.38, 0.57)	(−0.68, 0.18)

*, *p* ≤ 0.05; ASLR, Active Straight Leg Raise; TSPU, Trunk Stability Push-up; CMJ, Countermovement Jump; SLJ, Standing Long Jump; YBT, Y-balance test; FMS, Fundamental Movement Screen; CI, confidence interval.

## Data Availability

The data are available from Junjie Zhang but restrictions apply to the availability of these data. Therefore, the data are not publicly available.
